# Mapping Human Whole-Brain Structural Networks with Diffusion MRI

**DOI:** 10.1371/journal.pone.0000597

**Published:** 2007-07-04

**Authors:** Patric Hagmann, Maciej Kurant, Xavier Gigandet, Patrick Thiran, Van J. Wedeen, Reto Meuli, Jean-Philippe Thiran

**Affiliations:** 1 Department of Radiology, Centre Hospitalier Universitaire Vaudois (CHUV) and University of Lausanne, Lausanne, Switzerland; 2 Signal Processing Institute, Ecole Polytechnique Fédérale de Lausanne (EPFL), Lausanne, Switzerland; 3 Laboratory for Computer Communications and Applications, Ecole Polytechnique Fédérale de Lausanne (EPFL), Lausanne, Switzerland; 4 MGH Martinos Center for Biomedical Imaging, Harvard Medical School, Charlestown, Massachusetts, United States of America; Indiana University, United States of America

## Abstract

Understanding the large-scale structural network formed by neurons is a major challenge in system neuroscience. A detailed connectivity map covering the entire brain would therefore be of great value. Based on diffusion MRI, we propose an efficient methodology to generate large, comprehensive and individual white matter connectional datasets of the living or dead, human or animal brain. This non-invasive tool enables us to study the basic and potentially complex network properties of the entire brain. For two human subjects we find that their individual brain networks have an exponential node degree distribution and that their global organization is in the form of a small world.

## Introduction

Biological neuronal networks, and in particular the human brain, are remarkable natural systems capable of complicated patterns of behavior. This capability seems possible due to the combination of an enormous computational capacity given by a huge amount of neurons, and a highly evolved communication network [1]. To understand the mechanisms behind higher-level brain functions, a detailed study of the individual neural cells is clearly insufficient [2]; global functional and structural properties of such a complex system need to be considered as well [3]. This requires, first of all, a good knowledge of the network architecture of the entire brain. A graph representing the connectivity of the brain (henceforth called a ‘brain network’) can be analyzed at various scales. Probably the most obvious is at the neuronal level, where each neuron is a separate node in the graph and physical connections between neurons are reflected by the edges. This detailed view, however, is feasible only for the most primitive animals such as C. elegans with a brain made of 302 neurons [4]. A graph of the human brain consisting of 10^11^ nodes and 10^16^ edges is not only impossible to obtain with current techniques, but it also would carry a great deal of information that is irrelevant from the global organization point of view. We must therefore resort to a different level of granularity, where a node represents thousands or millions of neurons grouped together. Unfortunately, such available graphs are today limited to small post-mortem datasets (only 50–70 nodes) of rat [5], cat [6], [7] and monkey [8] brains, whereas larger datasets of animal and human brains are missing [9]. In the coming years, an immense effort will be needed to map at various scales and to create a large database of reliable information on the brain connectivity of higher order animals, especially of the human [10], [11].

Crick and Jones stated that *“Clearly what is needed for a modern human brain anatomy is the introduction of some radically new techniques”* [9]. In this paper we propose a methodology derived from diffusion MRI tractography [12]–[15] to map at a millimetric scale the structural white matter connectivity of the whole brain. The resulting network consists of nodes representing small areas of white matter–gray matter (WGM) interface, and weighted edges that capture long distance connection densities between these areas. The innovation it brings is fourfold. First, our methodology has a relatively *high resolution*; the resulting networks consist of thousands of nodes, which are 1–2 orders of magnitude larger than the networks currently available (thousands versus tens of nodes). This opens several innovative investigation possibilities. Mainly it allows us to study brain connectivity not only locally but also globally by characterizing the topological features of this large-scale network. Such global characterizations are essential for a better understanding of brain communication. Second, our approach is *non-invasive*. This allows us to study the topology not only of animal or post-mortem brains, but also, for the first time, of the living human brain. Third, for each subject we infer an *individual network* of the entire brain. This potentially allows us to compare individual subjects or groups of subjects, e.g., brains from healthy controls and from patients with clinical conditions. In contrast, the datasets available to date were collected part by part from a number of animals of the same species, and hence reflect a kind of “average” brain in the population. Fourth, our approach is *efficient*. It only requires performing an MRI scan on the subject (which takes about an hour or less depending on resolution and signal-to-noise ratio of the imaging system), and to process the data on a computer.

As an illustration of our approach, we analyze the basic brain graph properties of two healthy volunteers. In particular, we study a number of distributions derived for nodes (e.g., degree, strength) and edges (weight, length). We also answer some questions related to the topology, e.g., “Is the brain network a small world?”. With technology improvements, finer resolution and a better Signal-to Noise Ratio (SNR), or a deeper analysis of the network, more complex and accurate network characteristics will be accessible, thus potentially contributing to the answers of some key questions in neuroscience.

## Material and Methods

**Figure 1:**
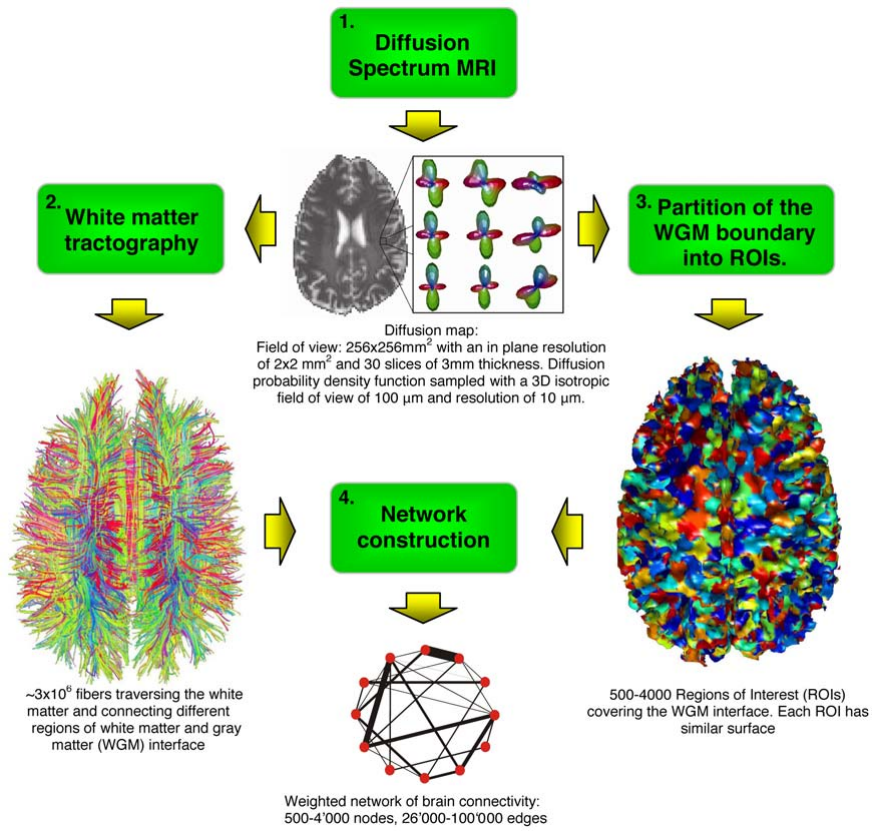
Mapping the network of brain structural connectivity with diffusion MRI is a process made of four steps. First, Diffusion Spectrum MRI (DSI) is performed on a subject or sample. This acquisition provides a 3D diffusion function at every location in the brain. This data set is called a *diffusion map*. It is shaped by the local tissue characteristics, in particular by the orientation of axonal bundles existing in the brain. Second, based on this map we generate a number of 3D curves (called fibers) that follow the path laid by the white matter axonal bundles. Third, independently from the previous step, we use a heuristic that partitions the brain white matter-gray matter interface into small areas of equal surface (called Regions Of Interest-ROIs) covering the whole cortex and deep cerebral nuclei boundaries. Finally, in the fourth step, we combine the output of steps two and three: the ROIs become nodes and the fibers are transformed into edges in the resulting graph. This graph estimates the density of white matter connections between any two regions of gray matter.

The path from diffusion MRI to a graph mapping brain connectivity is a four step process: (1) diffusion MRI acquisition, (2) white matter tractography, (3) white matter-gray matter interface partition into Regions Of Interest (ROIs) and (4) network construction. We present a general scheme of our methodology in Figure 1. Below we first describe each step illustrated with intermediary results. In the next section, we investigate some fundamental properties of the brain network inferred with our approach.

### Step 1: MRI acquisition

We use Diffusion Spectrum Imaging (DSI) [15], [16] . It is a diffusion MRI method that images the 3-dimensional diffusion function in every brain voxel and results in a 6-dimensional image called a diffusion map. This new method has, contrary to Diffusion Tensor MRI (DTI) , sufficient angular resolution to map accurately the diffusion with a non-Gaussian behavior. Accordingly it can see intra-voxel diffusion heterogeneity caused by crossing neuronal tracts, which is essential for an accurate mapping of axonal trajectories.

In the present experiment, after having obtained the informed consent of two healthy volunteers, two data sets are acquired at 3T with an Achieva (Philips, Einthoven, The Netherlands) MRI scanner using a diffusion weighted spin echo EPI technique [17], [18]. The timing parameters of the pulse sequence are TE/TR/Δ/δ = 154/3000/47.6/35 ms, maximum diffusion gradient intensity is 80 mT/m, yielding a maximal b value of 12000 s/mm^2^ [19]. The matrix size is 128×128 and the slice number is 30. The field of view is 256×256 mm^2^ and the slice thickness 3 mm, which yields a voxel size of 2×2×3 mm^3^. The classical DSI scheme we use goes as follows: diffusion-weighted images covering the whole brain are acquired for 515 different values of diffusion sensitizing gradient intensity and direction (i.e., different q-vectors) [20], comprising in q-space the points of a cubic lattice within the sphere of 5 lattice units in radius. We take **q** = *a* **q** *_x_*+*b* **q** *_y_*+*c* **q** *_z_*, with *a,b,c* integers and 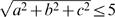, and **q** *_x_*, **q** *_y_*, **q** *_z_* denoting the unit diffusion sensitizing gradient vectors in the three respective coordinate directions. Next, we process these 515 images as follows. First, we reconstruct the 3D diffusion function, or Probability Density Function (PDF) at each brain location by taking the discrete 3D Fourier transformation of the signal modulus sampled in q-space. The signal is pre-multiplied by a Hanning window before the Fourier transformation in order to ensure a smooth attenuation of the signal at high |**q|** values. With this procedure and the above parameters, the PDF is sampled over an isotropic 3-dimensional field of view of 100 µm with a nominal isotropic resolution of 10 µm. The result, called a *diffusion map*, is a 6-dimensional image that associates a 3-dimensional diffusion function with every brain position voxel. From this map, at each voxel, we compute an Orientation Density Function (ODF) *Φ*(**u**), by projection of the PDF in the radial direction. If **u** is a 3D vector with |**u**| = 1, we define:
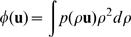
(1)where *p*(.) is the 3D PDF, *ρ* is the radius, *ρ ^2^dρ* is the 3D volume element and the integral is evaluated as a discrete sum over the available range *ρ*∈*[0,5]*. The ODF *Φ*(**u**) is a function defined on a discrete sphere and captures the diffusion “intensity” in every direction. It is evaluated for a set of vectors **u** *_i_* that are the vertices of a tessellated sphere that has a mean nearest-neighbour separation about 10°.

**Figure 2:**
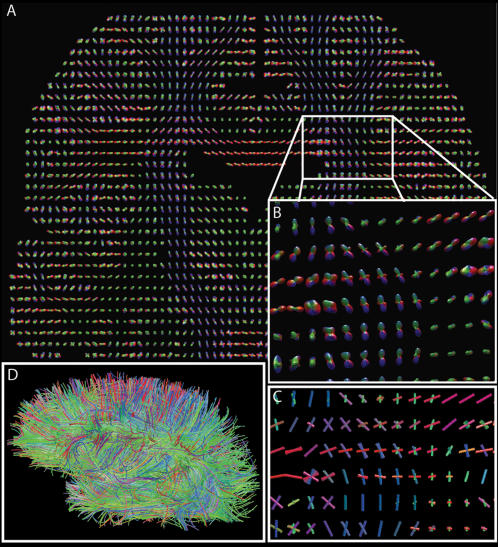
Tractography. A) The result of the “diffusion MRI acquisition” step. In every voxel of a coronal slide the Orientation Density Function (ODF) captures locally for every direction the diffusion “intensity”. B) Zoom in the centrum semi-ovale C) Each ODF is replaced by a set of vectors defining its local maxima. D) Fibers are computed following the local diffusion maxima; they are uniformly initiated over the whole brain white matter. See also Video S1 in Supporting Information.

In Figure 2 A and B we show a diffusion map, i.e., the ODF at every location in the brain. The ODFs are represented as deformed spheres with the radius proportional to *Φ*(**u**). The color-coding adds some more clarity, with blue codes for the cranio-caudal, red for left-right and green for antero-posterior direction.

### Step 2: White matter tractography

Tractography is a post-processing method that based on the diffusion map, constructs 3-dimensional curves of maximal diffusion coherence. These curves, called *fibers*, are the estimates of the real white matter axonal bundle trajectories [21]. We use a tractography algorithm specifically designed for DSI data to create a set of such fibers for the whole brain [15], [22] which is summarized below:

1. Detection of the directions of maximum diffusion. At each voxel, we define a set of directions of maximum diffusion as local maxima of *Φ*(**u**) (i.e., vectors **U** *_i_* such that *Φ*(**u** *_j_*)<*Φ*(**U** *_i_*) for all **u** *_j_* adjacent to **U** *_i_* in the sampled tessellated sphere (Figure 2 C).
2. Fiber computation. We initiate the same number of fibers for every direction of maximum diffusion in every white matter voxel. For example, in a voxel with 2 directions, we initiate 30 fibers along each direction, total 60. The starting points are chosen at random within the voxel. Next, from each such point we begin growing a fiber in two opposite directions with a fixed step of 1 mm. On entering a new voxel, the fiber growth continues along the direction of the vector **U** *_j_* (in the new voxel) whose orientation is the closest to the current direction of the fiber. If this results in a change of direction sharper than 15°/mm, the fiber is stopped. The growth process of a valid fiber finishes when both its ends leave the white matter. The resulting fibers can be interpreted as an estimate of the white matter axonal bundle trajectories (see Figure 2 D); in this article we use about 3 million initialization points of which only about one half to two third connect the white-gray matter interface and are retained (See also Vie).
3. **Filtering the edges.** In each of our data sets we have around 1.5 to 2 million fibers. For the graph of ∼1'000 nodes they translate into about 50'000 edges. The number of edges in the final network depends on the number of initialized fibers. To investigate network properties over a wider range of connection densities we devised two ways to filter edges by varying the number of initialized fibers or by taking into account the edge weight:
4.
  - **Random fibers**. Although for every data set we generate around 3 million fibers, this is not any special number. We could as well take 100 thousand or 10 million fibers. As presented in Figure S1 in Supporting Information, this would strongly affect the number of resulting edges. Therefore our first approach to limit the number of edges is to take a random subset of a given size out of our 3 million fibers, which boils down to reducing the number of fiber initialization. The study of the whole spectrum of fiber numbers gives us a better view than the study based on one, arbitrarily chosen number.
  - **Top-weight edges.** In the second method we consider the edges built based on all fibers, and delete only the edges with the smallest weights (according to some threshold). The heavy-tailed distribution of edge weights guarantees that we always retain most of the “edge mass” and reject only the edges with very small weights that are possibly the result of noise. Indeed noise may create aberrant diffusion maxima that in turn produce thin aberrant diffusion coherence paths across the data resulting into artefactual edges of small weight.

### Step 3: White matter-gray matter (WGM) boundary partition into ROIs

The goal of the third step is to partition the WGM interface in a number of areas that we call Regions Of Interest (ROIs). In this step we use exclusively the 3D mask of the brain WGM interface (i.e., the cortex and the thalamus for simplicity). The ROIs should be compact and of similar surface (counted in the number of voxels), which is a non-trivial task to achieve for the complex, strongly folded shape of the brain. For instance, a straightforward approach would be to partition this interface according to some 3D regular lattice [23]. Unfortunately, this approach results in large differences in ROI sizes-up to two orders of magnitude. Furthermore we do not want to partition the WGM into predefined areas like for example Brodmann's as they are too coarse (only about 50 to 55 areas) to analyze large scale network properties at high resolution. We have therefore developed a two-phase partitioning heuristic, as follows. First, we choose a WGM interface voxel at random and iteratively connect it to the neighbouring WGM interface voxels until it reaches the desired size; this structure becomes our first ROI. Similarly, we grow other ROIs, one by one, always starting near the ones that have already been created. We repeat this procedure until all the WGM interface is covered with ROIs. This gives us already quite a good partition, however, it can be easily further improved. Therefore, in the second phase of our heuristic we restart the ROI growth process. This time we grow all the ROIs simultaneously, starting from the centres of gravity of the ROIs found in the first phase. This results in a much better compactness of the ROIs with surface variations of less than 10% (See Figure S2 of Supporting Information). An example of the final result is shown in step 3 of Figure 1 (see also Video S2 in Supporting Information).

### Step 4: Network construction

Finally, in the fourth step, we combine the output of steps two and three and create the graph of brain connectivity. Every ROI constructed in step three becomes a node in the graph. We denote by ROI(*v*) the ROI that is associated with the node *v*. Two nodes *v* and *u* are connected with an edge *e* = (*v*, *u*) if there exists at least one fiber *f* with end-points in ROI(*v*) and ROI(*u*). For each edge *e* we define its length *l*(*e*) and weight *w*(*e*), as follows. Denote by *F_e_* the set of all fibers connecting ROI(*v*) and ROI(*u*) and hence contributing to the edge *e*. The *length l*(*e*) of the edge *e* is the average over the lengths of all fibers in *F_e_*, i.e., *l*(*e*) = 1/|*F_e_*|⋅Σ*_f∈Fe_l*(*f*), where *l*(*f*) is the length of fiber *f* along its trajectory. The *weight w*(*e*) captures the connection density (number of connections per unit surface) between the end-nodes of the edge *e*, and is defined as *w*(*e*) = Σ*_f∈Fe_*1/*l*(*f*). The correction term *l(f)* in the denominator is needed to eliminate the linear bias towards longer fibers introduced by the tractography algorithm. Indeed let us assume that an axonal bundle *b* exists in reality and has a length *l*(*b*). The tractography algorithm starts in some voxel of the white matter and follows the most probable direction of a bundle. If it happens to start in a voxel that is traversed by the bundle *b*, the algorithm follows *b* until it reaches the white matter boundary. As every voxel in the white matter is chosen as a starting point the same number of times, the longer the bundle *b* is, the more voxels it traverses and the more often it is followed by the tractography algorithm, introducing a linear bias that must be corrected.

ROI size is a parameter of our methodology. On the one hand, a natural lower limit for this size is one voxel of the WGM interface. However, we prefer to combine at least several voxels into one ROI to be sure to have a representative number of fibers connecting this ROI to the rest of the brain. On the other hand, taking ROIs that are too large results in a network of insufficient resolution and of trivially small size. In our simulations we set the ROI size to between 8 and 64 voxels of WGM interface. This results in a weighted network of between 500 to 4000 nodes representing small areas of WGM interface between ∼250 mm^2^ (64 voxels/ROI) and ∼30 mm^2^ (8 voxels/ROI), respectively. This graph has between 25'000 to 100'000 edges that represent axonal bundles of millimetric or centimetric diameter. For simplicity, in the remainder of this text we analyze graphs of about 1'000 nodes. In particular, |*V_1_*| = 1'013 nodes and |*E_1_*| = 47'217 edges for suject 1, and |*V_2_*| = 956 and |*E_2_*| = 50'199 for subject 2**.** The two graphs were built based on about |*F*| = 3 million fibers generated by the tractography algorithm. Results obtained for other granularities, from |*V*| = 500 to 4'000 nodes, are qualitatively similar (see Figure S3 of Supporting Information).

## Results and Discussion

Once the network constructed, several graph statistics characterizing the architecture of the network can be computed and examined.

### Node statistics

**Figure 3:**
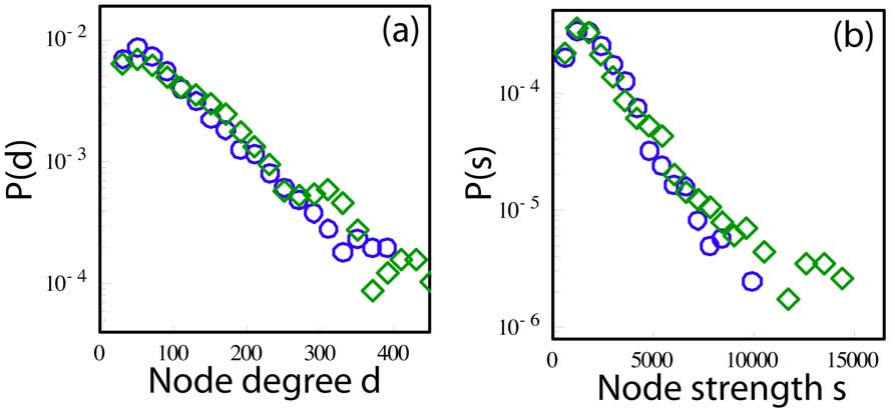
Basic characteristics of nodes in the graph of brain connectivity. *P(d)* [*P(s)*] is the probability that a randomly chosen node has the degree [strength] equal to *d* [*s*]. The node degree distribution (a) and node strength distribution (b) are lin-binned and plotted in log-lin scale. Color code: subject 1 (blue circles), subject 2 (green diamonds)

We first turn our attention to the nodes of our graph. A basic characteristic of a node *v* is its *degree*, i.e., the number of edges incident on *v*. Many complex networks such as the World Wide Web, Internet, protein networks, ecological networks or cellular networks, have been shown to follow a heavy-tailed node degree distribution [24]. In other words, they have a very significant number of high degree nodes, called hubs. As such networks, also called “scale free”[24], are characterized by relatively short distances between any two nodes and by high robustness to random failures [25], they seem, at first sight, to be good candidates for brain topology. Surprisingly, we find in our dataset that this is not the case. In Figure 3, we plot the node degree distribution (a), and a closely related node strength distribution (b). (The *strength s* of a node *v* is the sum of weights of all edges incident on the node *v*, *s*(*v*) = Σ*_e:v∈e_w*(*e*)[26].) Although these distributions cover more than two decades, they are roughly linear in the log-lin scale, which indicates their exponential tail. This is probably the first time that a claim about the node degree distribution of cortical structural connectivity mapped at high spatial resolution can be made. The networks available and studied to date [27], [28] are simply too small to judge if their node distribution is exponential, heavy tailed, or yet different. It is worth mentioning that in contrast to structural analyses, some functional brain networks have been described as scale-free [29].

A closer look at node degrees suggests that, from a developmental and energy optimization point of view, hubs do not seem to be favored. This was suggested by [30] who modeled the development of frontal macaque cortex by a spatially embedded growing graph where preferential attachment occurs as an exponentially decaying function of spatial distance and growth limited in space. Amaral et al. [31] modeled network growth where the node degree expansion is attenuated through node aging and energy limitations. These two models, like our measurements, resulted in networks with an exponentially decaying distribution. Furthermore, it is quite unlikely to find hubs in the gray matter, because we know that the neuronal density does not change over orders of magnitude across the cortex [32], [33].

### Edge statistics

**Figure 4:**
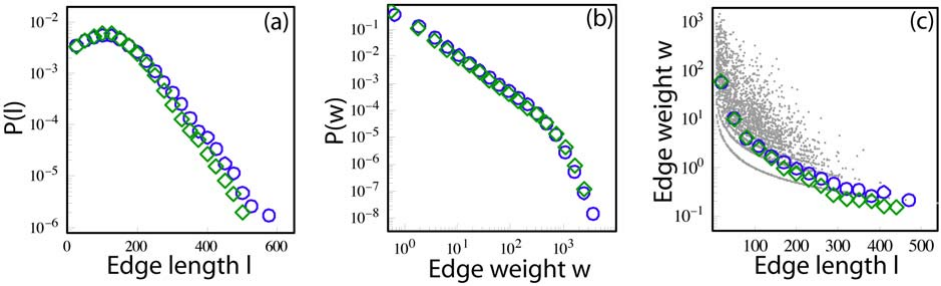
Basic characteristics of edges in the graph of brain connectivity. (a) The distribution of edge lengths *l* in log-lin scale, lin-binned. (b) The distribution of edge weights *w* in log-log scale, log-binned. (c) Scatter plot of *w* vs *l*. The symbols are lin-binned average values for subject 1 (blue circles) and subject 2 (green diamonds).

The edge length *l* distribution decays exponentially (Figure 4a), indicating that the number of long connections is small. The edge weight *w* distribution is much broader and close to heavy-tailed (Figure 4b). Therefore, there are a significant number of very strong connections that are predominantly short as demonstrated in Figure 4c. This observation is in agreement with the results of other complementary studies on the organization of the brain that suggest that brain favors, with some intriguing exceptions, locally dense communication and minimizes the number of long distance connections [34]. For instance, by examining many alternative arrangements of macaque pre-frontal cortex, [35] showed that the layout of cortical areas minimizes the total lengths of the axons needed to join them. A similar observation was made by [36] about the intrinsic gray matter connectivity of mice where the volume fraction of axons and dendrites seems close to optimal. The work of [37] indicate that there is an evolutionary conserved scaling of the volume of the white matter as the 4/3 power of the volume of the gray matter, which can be explained by the fact that global geometry of the cortex minimizes the average length of the long-distance fibers while keeping the average connection density of long-distance connection fibers constant. However recent reports suggest the organization of neural networks is not only shaped by the minimization of total wiring length. Multiple constraints seem to be involved, not only wiring length but also the average number of processing steps (related to the average distance between node) [38].

### Network topology

Having examined separately the distributions of nodes and edges, we now discuss the topology of the graph itself. An interesting question one can ask is: “Is the brain a small world?”. The more formal definition of a small world graph involves two metrics, *clustering coefficient c* and *average shortest path length* <*sp*>. We follow [39], who define the clustering coefficient *c* as the probability that two randomly chosen neighbors of a node are also direct neighbors of each other, i.e., *c* = 1/|*V*|⋅Σ*_v∈V_c*(*v*), where *c*(*v*) is the number of edges interconnecting the neighbors of the node *v* divided by the number of all possible edges. The average shortest path length <*sp*> is the average distance between any two nodes in graph. If the graph is disconnected, only the largest connected component is considered. A graph is called a *small world* if it has (i) a clustering coefficient much greater than that of equivalent random graphs and (ii) the average shortest path length <*sp*> is comparable with that of a random graph with the same number of nodes and edges [39].

There are two issues that we have to address before we attempt to decide if our graph *G* of brain connectivity is a small world. First, *G* is weighted. As there exists no standard way of generalizing the clustering coefficient to weighted graphs [see e.g. [40], [41]] and it is not obvious how to interpret edge weights when computing the average shortest path length, we have decided to treat every edge equally and apply the classic unweighted approach [39]. Second, the number of fibers that are initiated during tractography determines the density of graph G. In order to explore the effect of connection density on our results, we exclude some of the edges by applying the two filtering techniques described above.

**Figure 5:**
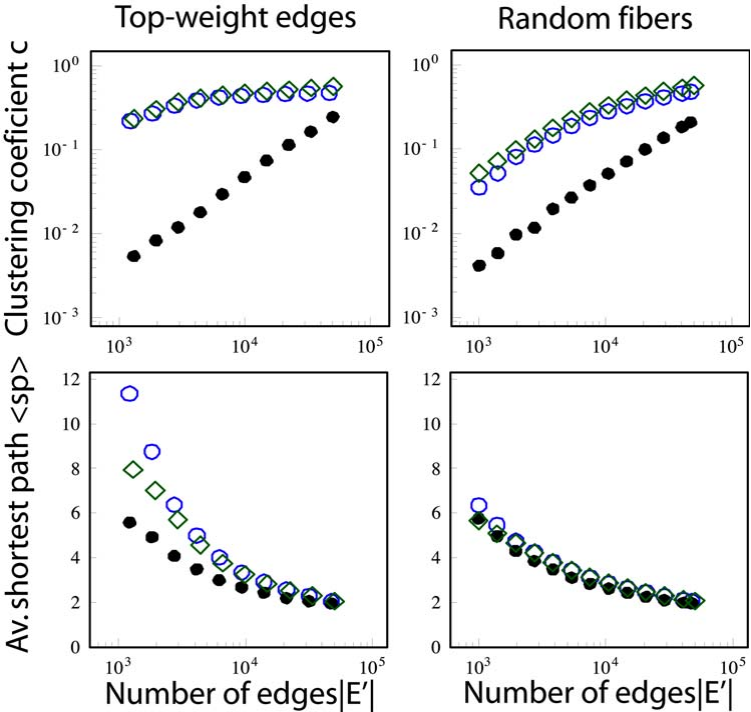
Average shortest path <*sp*> and clustering coefficient *c* as a function of the number of edges in the brain graph |*E'*|. The edges are chosen from the set of all edges *E* either giving the priority to the edges with high weights (“Top-weight edges”, left column), or based on a random subset of fibers (“Random fibers”, right column). As a reference we take a random graph with the same number of nodes and edges, and the same degree distribution. Color code: subject 1 (blue circles), subject 2 (green diamonds), random graph reference (black filled circles). The results are averaged over 10 realizations of the “random fibers” filtering and random graphs; the confidence intervals (not shown) are comparable with the symbol size.

We present the results in Figure 5. As a reference we take a random graph not only with the same number of nodes and edges (as proposed in [39]), but also with the same degree distribution as the brain graph. This graph was generated with the rewiring technique described in [42]. Preserving the degree distribution allows us to rule out this factor from the set of possible reasons of observed differences between the brain and the reference topology. For any number |*E'*| of edges remaining after the filtering, the graph of brain connectivity has a much higher clustering coefficient than the corresponding random graph; this is especially well pronounced for the “Top-weight edges” graph. At the same time their average shortest path lengths <*sp*> are comparable. Hence our measurements suggest that the small-world model fits the brain network. Indeed, the small-world topology seems well suited for the neuronal network of the brain when thinking in evolutionary and developmental terms. This is because it is a good compromise between full connectivity, which would be very costly in terms of wiring (i.e., brain volume) and power supply [43], and a lattice topology that impairs massively long distance communication. Furthermore, the combination of high local clustering and small average shortest path length with efficient neural coding [44] allows for a distributed computing where only a small fraction of local intense computation needs to be transmitted to distant regions, which may be sufficient for synchronous brain activity [45]. The small-worldness of the brain network was already advocated before, based on relatively small networks (50-70 nodes) resulting from post-mortem tracing studies in rat, macaque monkey and cat brain regions [27], [46], [47]. In contrast, the approach presented in this article provides, for the first time, evidence for the presence of small-world topology in the structural connectivity of the human cerebral cortex. Moreover, the one to two orders of magnitude higher resolution resulting from our method (thousands vs. tens of nodes) increases the confidence we have in the derived statistics.

### Intra- and inter-individual network differences

In order to test the robustness of our methodology and because of uncertainty about the ideal number of nodes for the presented methodology, we measured the brain network at 4 different node resolutions on data set 1 (see Figure S3 of Supporting Information). We notice that for scales varying between 500 and 4000 nodes and 25'000 and 100'000 edges respectively, the global network topology is preserved. This is a range of scales that matches the sensitivity of the method, as we do not expect to be able to accurately map tracts smaller than several milimeters in diameter, which is presently the size of our ROIs. Pushing the network “resolution” higher by increasing the number of nodes and reducing the surface area of the ROIs would increase the quantification noise (limiting the number of fibers per ROI), which ultimately would destroy the information contained in the network model. On the other hand, increasing the ROI size, limits the precision of the mapping, potentially grouping together pieces of gray matter that are functionally different. At the scale we use in this study, we expect that the chance that ROI overlaps several critically different cortices is not higher than the inaccuracy related to the matching of template atlas on our data. Notwithstanding the advantage with a fine grain method to always be able to group arbitrarily sets of nodes in order to study connectivity patterns between for example well known functional or anatomical areas like Brodmann's.

While basic connectivity parameters differed slightly for data sets 1 and 2 (see Table 1), the global properties are quite similar. The differences that we observe in Figures 3, 4, 5 may or may not reflect the individual properties of the subjects. Clearly, more experiments and studies are needed to be able to address the issue of between-subject variability with a high level of confidence. We plan to address these issues in our future work.

**Table 1:** Network construction parameters for data set 1 and 2

	Data Set 1	Data Set 2
**Number of nodes**	**1013**	**956**
**Number of fibers**	**1'677'892**	**1'833'794**
**ROIs area**	**1.28 cm^2^**	**1.44 cm^2^**
**Number of edges**	**47'217**	**50'199**

The question of investigating structural network deteriorations in diseased populations like schizophrenics or demented patients is challenging and should be addressed in the future [48], [49]. The first issue is to decide on the most representative measure of tract degradation. Should we use the connection density as presented in this article? Or are differences in connectivity better captured through the use of the mean fractional anisotropy or the diffusion trace along a connection as is done in several DTI studies [50], [51]? If we want to capture the global network topology, the only requirements are to use the same MRI acquisition and simulation parameters, such as the number of nodes and the way fibers are initiated. The task becomes much more challenging if our goal is to perform an edge-by-edge comparison. The problem is twofold. First, we have to match the nodes across subjects. This requires precise cortical registration tools that work with a sub-centimetric precision. Second, identifying significant changes when testing thousands of edges at once will either require a large cohort or strong network changes, as the significance threshold needs to account for multiple testing.

Although our methodology yields promising results, we need to keep in mind that there are some steps prone to various kinds of noise and distortions whose effect is difficult to evaluate. First of all, we work at a given level of granularity. The spatial and angular resolution of our diffusion MRI experiment is limited, which makes it difficult to tell much about submillimetric fiber tracts and crossing axonal bundles separated with angles smaller than 20°. The ROIs have a given size, which automatically groups tens of thousands of neurons into a single node. Noise is also introduced during the MRI acquisition, and the tractography algorithm might not perfectly model the relationship between water diffusion and axonal orientation. Although all these points are constantly being improved, there will always remain a huge discrepancy between our constructed graph and the real neuronal network made of 10^11^ neurons and several orders more connections.

### Quality control

Nevertheless, diffusion MRI tractography is a widely used and accepted method to map axonal bundle trajectories. Furthermore it was validated experimentally to large extent in the case of DSI. First, [21] show that the ODFs produced by DSI match accurately the fiber orientations in a phantom and follow accurately the optic tracts in the rat. Second, [52] validate the method in the monkey by comparing DSI tractography with histological autoradiographic tracing over 10 association tracts. This study shows a remarkable agreement of results between two fundamentally different techniques. In addition to these general arguments, we have also tested our particular data set. Figure 6 presents a qualitative impression of the type of data revealed by our method, by showing the connectivity of part of the cortical visual system. More specifically we investigate the well-studied connections between areas V1, V2, V3, V5 and the lateral geniculate body [53]–[55]. The different visual areas were identified manually based on the gyral anatomy and consist each of a set of ROIs. A set of well known connections was identified for the purpose of illustrating the tractography method without claiming to be a detailed study of the visual system which would require a functional retinotopic mapping of the visual areas and an extensive search and study of the individual fiber bundles. Our data not only reveals intermediate length connections between V1 and V2 or between V2 and V3, but also the well known long range connections such as i) the optic radiation–linking the lateral geniculate body to V1 , ii) V1 homotopic callosal projections, which are connections that take actually their origin more at the junction between V1 and V2 [56] and iii) V2–V5. Furthermore, the weights of these edges are by far higher than the corresponding median weights over the whole brain (see Figure S4 in Supporting Information). This gives a good level of confidence that the observed visual connections are not caused by some random effect.

**Figure:**
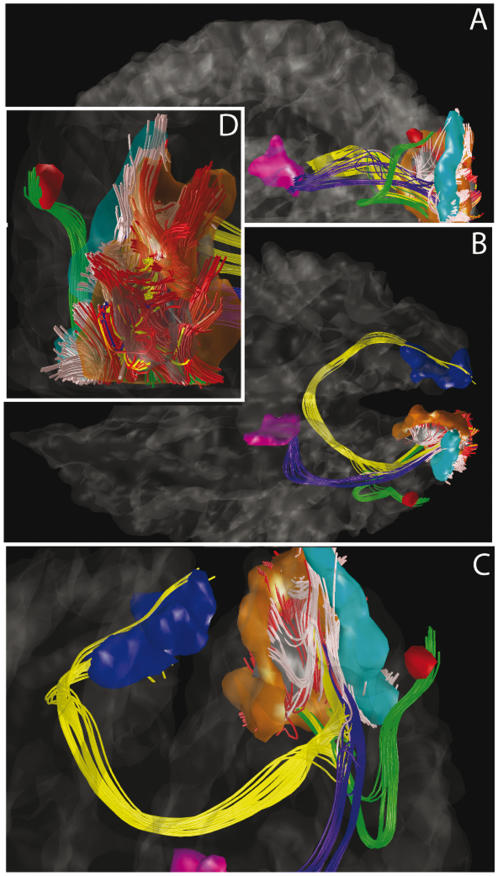
Visual system white matter connectivity derived from tractography: Views from the left (a), from the top (b), zoomed anterior (c) and posterior (d). White matter–gray matter interfaces: Magenta = posterior part of the thalamus, blue and gray = right and left V1, orange = V2, cyan = V3, red = V5. Fibers: yellow = homotopic V1, red = V1–V2, white = V2–V3, green = V2–V5, blue = lateral geniculate body–V1. See also Video S3 in Supporting Information.

### Conclusion

In this article we have proposed a methodology for mapping networks of structural connectivity in the brain. Our approach is non-invasive, efficient, individual and of relatively high-resolution. For the first time we can globally characterize brain connectivity with individual tract properties or network statistics in an individual living subject. Based on the analysis of two healthy subjects we found that the graph of the human brain is a small world, but not a scale-free network. Large new areas of application are foreseen; in basic neuroscience our technique may contribute to the discovery of the general principles regulating communication, evolution and development of the brain; in clinical neuroscience it may shed new light into diseases of disorders that involve disruptions of anatomical brain connectivity.

## Supporting Information

Figure S1 The number of edges in the resulting graph as a function of the number of fibers connecting two points in the gray-white mater interface. The straight line represents the y = x relation.
(0.05 MB TIF)

Figure S2 Histograms of ROI sizes for the number of ROIs ranging from N = 506 to 4052 in subject 1. One voxel translates to about 4 mm^2^.
(0.94 MB TIF)

Figure S3 The results generated for all four considered scales in subject 1. The symbols in the last two rows are (as in the main paper): blue circles-“Top-weight edges”, red triangles-“Random fibers”, and black disks-“Random graph”.
(6.92 MB TIF)

Figure S4 Comparison of edge weights inside the visual system with the rest of the brain. Each box plot represents all edge weights in the brain of similar white matter length. The big black dot represents the weight of the considered connection, namely V1-V2, V2-V3, V2-V5, as well as the connections between the lateral geniculate body and V1 (LGB-V1), and between left and right V1 areas (V1^left^-V1^right^). Each connection is compared with the other connections in the brain of same white matter length as short connections are usually denser that long ones. The considered connections in the visual system are largely above their respective medians (horizontal line in within each box, whiskers represent 5th and 95th quantiles).
(0.39 MB TIF)

Video S1 Whole brain tractography result in subject 1.
(5.23 MB MPG)

Video S2 Partition of the white-gray matter interface in approximately 1000 ROIs.
(2.01 MB MPG)

Video S3 Connections between different visual areas.
(1.65 MB MPG)
