# Supplementary figures and images for: Mapping Human Whole-Brain Structural Networks with Diffusion MRI
Source: PLoS One. 2007 Jul 4;2(7):e597. doi: 10.1371/journal.pone.0000597 (PMC1895920; doi:10.1371/journal.pone.0000597)

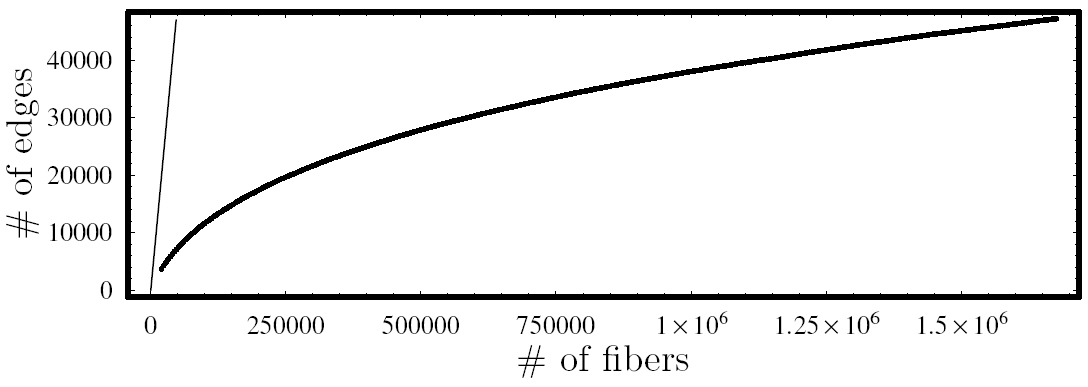

Supplement: Figure S1 — The number of edges in the resulting graph as a function of the number of fibers connecting two points in the gray-white mater interface. The straight line represents the y = x relation. (0.05 MB TIF) [file pone.0000597.s001.tif]

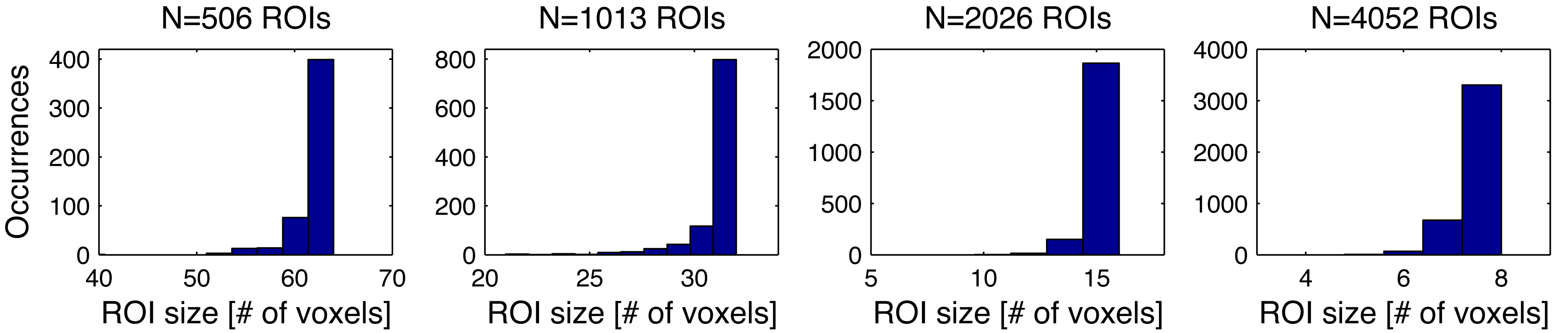

Supplement: Figure S2 — Histograms of ROI sizes for the number of ROIs ranging from N = 506 to 4052 in subject 1. One voxel translates to about 4 mm2. (0.94 MB TIF) [file pone.0000597.s002.tif]

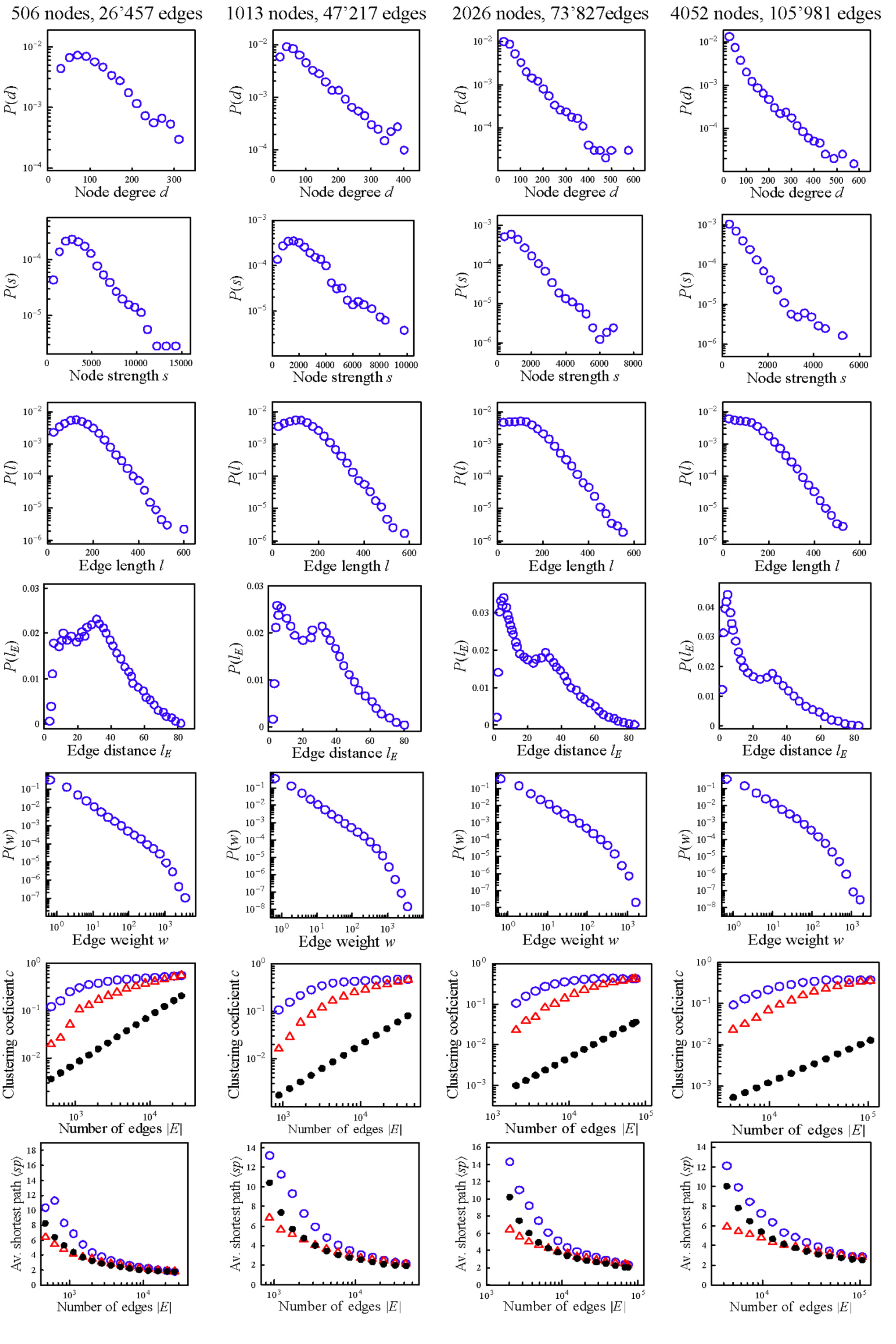

Supplement: Figure S3 — The results generated for all four considered scales in subject 1. The symbols in the last two rows are (as in the main paper): blue circles-“Top-weight edges”, red triangles-“Random fibers”, and black disks-“Random graph”. (6.92 MB TIF) [file pone.0000597.s003.tif]

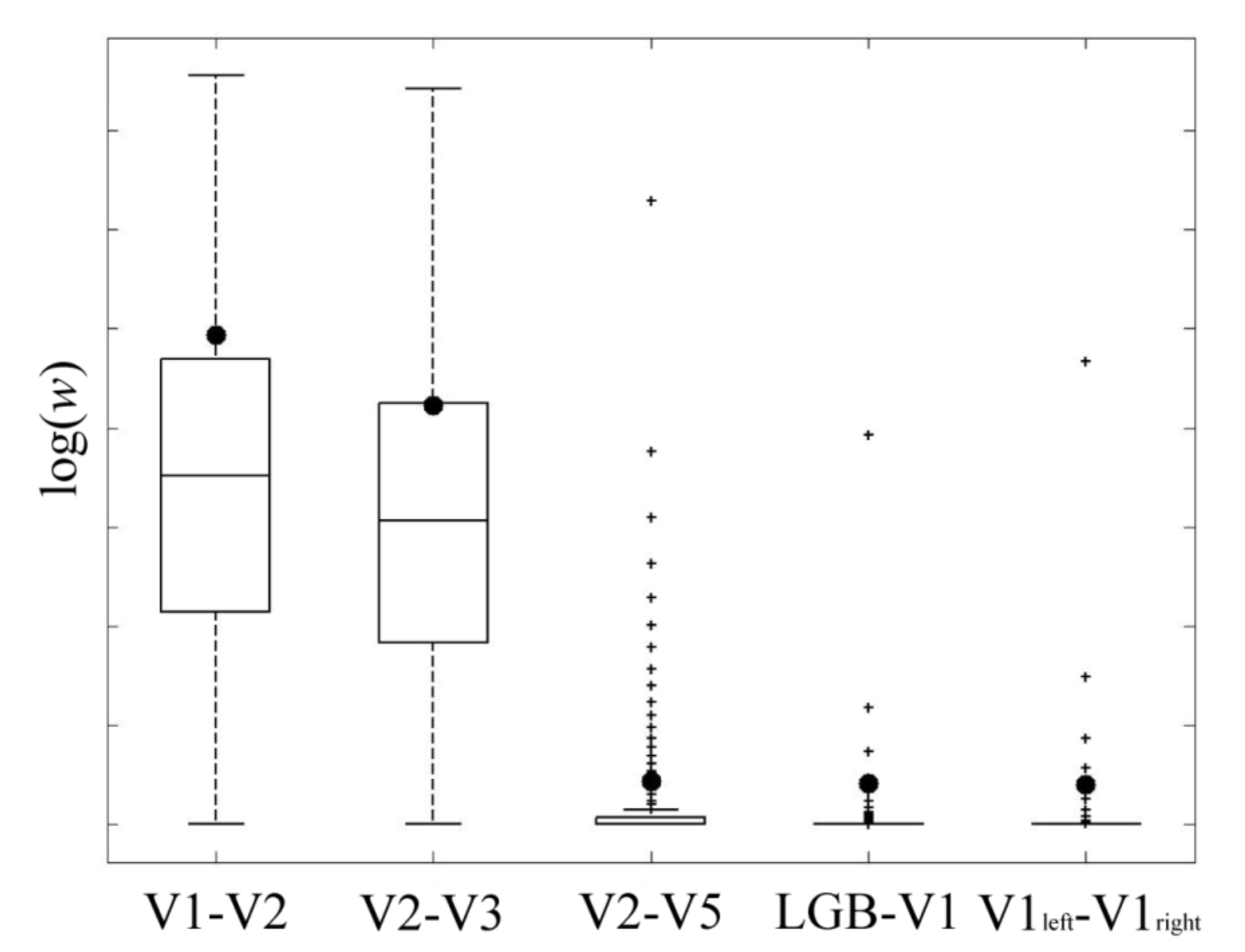

Supplement: Figure S4 — Comparison of edge weights inside the visual system with the rest of the brain. Each box plot represents all edge weights in the brain of similar white matter length. The big black dot represents the weight of the considered connection, namely V1-V2, V2-V3, V2-V5, as well as the connections between the lateral geniculate body and V1 (LGB-V1), and between left and right V1 areas (V1left-V1right). Each connection is compared with the other connections in the brain of same white matter length as short connections are usually denser that long ones. The considered connections in the visual system are largely above their respective medians (horizontal line in within each box, whiskers represent 5th and 95th quantiles). (0.39 MB TIF) [file pone.0000597.s004.tif]
